# Structural model of the relationship between physical activity and students’ quality of life: Mediating role of body mass index and moderating role of gender

**DOI:** 10.1371/journal.pone.0273493

**Published:** 2022-08-26

**Authors:** Mahdieh Hoseini, Samaneh Bardoon, Afsaneh Bakhtiari, Hajar Adib-Rad, Shabnam Omidvar

**Affiliations:** 1 Student of Research Committee, Babol University of Medical Sciences, Babol, Iran; 2 Department of Health, Social Determinants of Health Research Center, Health Research Institute, Babol University of Medical Sciences, Babol, I.R.Iran; 3 Department of Nursing and Midwifery, Infertility and Health Reproductive Research Center, Health Research Institute, Babol University of Medical Sciences, Babol, I.R.Iran; 4 Department of Nursing and Midwifery, Social Determinants of Health Research Center, Health Research Institute, Babol University of Medical Sciences, Babol, I.R.Iran; Shahrood University of Medical Sciences, ISLAMIC REPUBLIC OF IRAN

## Abstract

**Background:**

As a country’s future leaders and pioneers, University students must live with healthy habits. In order to achieve a healthy lifestyle, Physical activity and Quality of Life can serve as suitable indices to study. The purpose of the study was to clarify how physical activity (PA), Body mass index (BMI) and gender relate to the quality of life (QOL) of students of Medical Sciences University by using a structural equation model.

**Methods:**

The research was a cross-sectional study. The number of participants was 225 students of the University. The participants answered three questionnaires, including Demographic, International Physical Activity Questionnaire (IPAQ short form), and Quality of Life Questionnaire (SF-12), BMI was calculated by anthropometric measures, as well. The Structural equation model (SEM) method was employed. The Fitness of the proposed pattern was measured using the following indexes: chi-square/degree of freedom ratio (CMIN/DF), Normed Fit Index (NFI), comparative fit index (CFI), the goodness of fit index (GFI), and standardized root mean squared residual (SRMR). In the analysis the significant level was considered as P < 0.05.

**Results:**

PA (r = -0.726, P<0.001) and QOL (r = -0.405, P<0.001) have significantly inverse relationship with BMI, whereas the QOL and PA were proven to be positively related (r = 0.357, P<0.001). Moreover, the results signify gender as a moderator in the relationship between PA and QOL (Δχ^2^ (10) = 19.903, P = 0.030) and also the mediatory role of BMI among students. BMI affects the QOL in men (P < 0.001, β = -0.307) more in compare to women women (P = 0.324, β = -0.158).

**Conclusion:**

Study findings supported the research hypothesis. Gender exhibited moderating role in the relationship between PA and QOL, considering the mediating role of BMI.

## Introduction

Physical activity can be defined as body movement escalating into a considerable rise in energy consumption, in compare to resting. Appearing in many forms, the amount of physical activity is affected by its frequency, intensity, and duration. Despite the great importance of the physical activity to the well-being of individuals, it is reported that almost 30% of women and 25% of men are physically inactive; in other words, more than one- quarter of the world’s population is inactive [1, 2]. It is reported that physical inactivity can be considered a major risk factor affecting the mortality of various diseases and is associated with almost 3.2 million deaths, annually [3]. Physical inactivity leads to weakened general health and is hence being recognized by the medical and health community, rapidly [4]. It is known that PA has a positive impact on various health indicators [5, 6]. Regular physical activity encourages a better QOL, as well as reduced chances of morbidity and mortality in diseases [7, 8]. PA can be effective on multiple aspects of individuals’ QOL [9–11]. Researchers suggest that individuals with a higher level of PA tend to have a better score on QOL when compared to people with less physical activity [12–15]. In addition to that, physical activity also affects an individual’s physical, psychological as well as emotional health [16]. This can be seen in different stages of life [17]. Study by Javadivala exhibited that there was a significant and positive relation between PA and QOL in menopausal women [18]. The obesity trends give rise to estimations such that by the year 2030, 65 million and 11 million obese adults will be living in the US and the UK, respectively [19]. The prevalence of obesity has been reported to be ranged from 22 to 48% in eastern countries [20–22]. WHO has reported that almost 2 billion adults are overweight out of which 650 million suffered from obesity. This report dates back to 2016, following a Global action plan on physical activity which has been planned through the years 2018 to 2030 to encourage a healthier lifestyle with increased physical activity [2]. The World Health Organization (WHO) aims to diminish the prevalence of physical inactivity by 15% by the year 2030 [23]. The WHO explains that the positive side of the concept of health, is a positive attitude of society towards the improvement and upkeep of health as the main condition for social well-being [24].

QOL consists of multiple domains. It can be associated with positive feelings such as happiness, wealth, success, health and satisfaction [25]. QOL can be assumed as a crucial tool in health care. The World Health Organization Quality of Life assessment (WHOQOL) group has defined QOL as "an individual’s perception of their position in life in the context of the culture and value systems in which they live and in relation to their goals, expectations, standards and concerns" [26].

Sedentary behavior is a crucial health issue in children and adolescents, which is intensified with age [27]. University students often experience a sitting time of over 9 hours a day which could set obesity in motion [28]. It is assumed that a large number of college students follow an unhealthy lifestyle (Alcohol over-consumption, tobacco use, sedentary behavior, etc.) [29, 30]. The level of physical activity starts to significantly decrease during adolescence, and the period of life in which the turn from adolescence into adulthood happens is very important [31]. Several studies have shown that physically active people have a better quality of life than inactive people. However, the improvement in quality of life was only observed in participants with high levels of physical activity [13, 14, 32]. There are many other factors which have effects on individuals’ quality of life. A study found that gender was not a determinant of correlations between PA and QOL [33] and it was contrary with the results of other studies [34, 35]. It seems that the role of gender in the relationship between PA and quality of life is still poorly understood. In addition, in two US population studies that used single-item life satisfaction measures, obesity was associated with decreased levels of life satisfaction [36, 37], while a Danish epidemiological study, controlled for several lifestyle factors, including body mass index (BMI), found a positive and independent association between self-reported physical activity and satisfaction in life [38]. Therefore, it was assumed that there might be a relationship between PA and QOL, with mediating or moderating effects of BMI and gender.

## Materials and methods

The present study was a cross-sectional study. The participants included all students studying at the Babol University of Medical Sciences (BUMS). Multistage sampling was applied to select the subjects. All the university majors were 22 such as medicine, dentistry, nursing and midwifery, public health etc. were initially considered as clusters, and then among them ten majors were selected by simple random sampling. In the next step, participants were selected conveniently from the chosen clusters. We invited 25 students from each majors to participate in the study. The response rate was 90 percent and finally the data of 225 students who provided informed consent for study participation, was included in the study.

203 participants were initially assumed sufficient for this study to achieve a power of 80% at alphas level of 0.05 based on correlation coefficient of 0.196. In addition to that, a 10% drop-out rate was assumed, resulting in a sample size of 225.


$$n={\left(\frac{Z_{1-\frac{\alpha }{2}}+Z_{1-\beta }}{\frac{1}{2}\ln\frac{1+\gamma }{1-\gamma }}\right)}^{2}+3$$

Inclusion criteria were being a student at BUMS. Exclusion criteria were disability imposing limitations to physical activity, diagnosis with diseases with an effect on BMI (Diabetes, Thyroid, etc.), depression and other similar psychological disorders, the experience of a sorrowful incident in the past 3 months limiting physical activity or hindering the personal quality of life.

### Ethics approval and consent to participate

The study was approved by the Research Ethical Committee of Babol University of Medical Sciences (MUBABOL.HRI. REC.1399.061). Written informed consent was taken from all the participants.

### Instruments

We used three questionnaires including:

1. Demographic characteristics questionnaire: including age, marital status (single, married), residential status (Dormitory, house along with others, house alone), gender (female, male) and occupation (yes, no), family financial status (low, middle and high), etc. For assessing family financial status participants answered a question: (what is your family financial status?)Anthropometric measurements: The BMI was calculated through the division of weight in kilograms by the square of height, measured in meters. The weight was measured through a portable electronic scale, with an accuracy of 0.1 kg, and the height was assessed using a stadiometer with an accuracy of 0.1 cm. WHO classification was used to classify the participants’ BMI [39].

2. International Physical Activity Questionnaire (IPAQ): The short version of the International

Physical Activity Questionnaire IPAQ (IPAQ-SF) was employed to assess the physical activity of individuals. The IPAQ-SF is a scale with 7 items. It includes questions on physical activities related to work, transportation, housework, and leisure time during the past 7 days. Metabolic equivalents (METs)-min were calculated by multiplying the duration of activity in minutes by the coefficient of the activity level (1.3 for sitting, 3.3 for walking, 4 for moderate, and 8 for vigorous activities) [40]. The total Metabolic Equivalents (METs) estimation was utilized to divide the participants into different groups. The division was such that participants experiencing moderate activity, vigorous activity, or walking during the past 7 days, with a score under 600 MET-min/weeks were placed in the low physical activity group. Similarly, participants scoring between 600 and 3000 MET-min/week were placed in the moderate physical activity group, and those with over 3000 MET-min/week were placed in the high physical activity group. This version of IPAQ has been proven to have validity and reliability with co-relation factors of 0.77 and 0.95 [41–43]. The Persian version of this questionnaire has been reported to have Alpha Cronbach 0.7 and reliability was assessed by test re-test [44].

3. QOL questionnaire SF-12 is a self-reported questionnaire including 12 items from the original 36 items questionnaire (SF-36). The 12 questions assess 8 domains physical functioning 2 items, role physical 2 items, bodily pain 1 item, general health 1 item, vitality 1 item, social functioning 1 item, role emotional 2 items and mental health 2 items. The response categories for items ranged from 2 to 6 point scales and raw scores for items are ranging from 1 to 6. After recoding raw scores for some items (that are Bodily Pain, General Health, Vitality, and one item from Mental Health); then the raw scores could be transformed in order to provide eight scale scores each ranging from 0 (the worst) to 100 (the best). The physical component summary score (PCS) from the Physical Health domain and the mental component summary score (MCS) from the Mental Health domain the scores could range from 0 to 100 with higher scores indicating a better QOL. Montazeri et al have evaluated the validity of the Persian version of this questionnaire [45].

We tested two hypotheses:

1. The effects of physical activity on quality of life are mediated by BMI.

2. The mediated effects of physical activity on QOL through BMI are moderated by gender.

### Statistical analysis

#### Research structural model

To assess the suggested pattern between physical activity and students’ quality of life, we conducted structural equation modeling (Maximum likelihood estimation method) using AMOS version 24. Index fitting was performed using Chi-square and Chi-square degree of freedom ratio index (CMIN/DF), Parsimonious Normed Fit Index (PNFI), Comparative Fit Index (CFI), Parsimonious Comparative Fit Index (PCFI), Incremental Fit Index (IFI), Goodness-of-Fit Index (GFI) and Root-Mean-Square Error of Approximation (RMSEA). In addition to that, we have applied multi-group analysis through measured weight models comparison to study the moderating effects of gender, as a variable. To assess the research hypotheses, the assumptions were studied, prior to the application of the structural equations method. We have utilized the univariate and multivariate data distribution to analyze the normal and outlier data, separately. The test of univariate and multivariate normality and multicollinearity indicated the data were normally distributed (Mahalanobis d-squared p values more than .001) and multicollinearity was not an issue (VIF <5) [46].”

## Results

The present study comprised 121 (53.8%) females and 104 (46.2%) male participants, with an average age of 23.06±2.63 and the age range of 19 to 37 years. The results exhibited that 93.3% of the students were single, 13.8% were employed and 57.4% were in the average or poor economic status group. The results depict that the mean BMI (kg/m^2^), mean physical activity (METs), and the mean quality of life of the participants were 23.86±3.87, 2008.28±2703.1, and 42.39±3.68, respectively. Interestingly, 34.8% of the subjects were overweight or obese and 32.1% had low PA.

The results exhibited that physical activity levels have a significant relationship with gender, occupation, smoking, frequency of exercise, duration of exercise, and BMI (Table 1).

**Table 1:** Characteristics of the students and its’ association with physical activity levels (N = 225).

Variables		Total	Level of activity			P-value
			Low n (%)	Moderate n (%)	Vigorous n (%)	
Gender ^a^ n (%)	Female	121(53.8)	51(42.9)	53(44.5)	15(12.6)	< .001
	Male	104(46.2)	20(19.2)	59(56.7)	25(24)	
Marital ^a^ status n (%)	Single	210(93.3)	64(30.8)	108(51.9)	36(17.3)	.102
	Married	15(6.7)	7(50)	4(28.6)	4(21.4)	
Occupation ^a^ n (%)	Yes	31(13.8)	8(25.8)	11(35.5)	12(38.7)	.005
	No	194(86.2)	63(32.8)	101(52.6)	28(14.6)	
Family financial status ^a^ n (%)	High	96(42.6)	31(32.2)	45(46.8)	19(21)	384
	Middle & low	129(57.4)	40(31.1)	67(51.9)	21(17)	
Residential status ^a^ n (%)	Dormitory	95(42.2)	26(27.7)	47(50)	21(22.3)	.087
	House (along with others)	104(46.2)	31(30.1)	56(54.4)	16(15.5)	
	House alone	26(11.6)	14(53.8)	9(34.6)	3(11.5)	
Smoking ^a^ n (%)	Yes	24(10.7)	7(29.2)	8(33.3)	9(37.5)	.026
	No	201(89.3)	64(32.2)	104(52.3)	31(15.6)	
Alcohol drinking ^a^ n (%)	Yes	21(9.3)	3(14.3)	11(52.4)	7(33.3)	.070
	No	204(90.7)	68(33.7)	101(50)	33(16.3)	
Sleep duration ^a^ (hour) n (%)	>7	68(30.2)	27(40.3)	32(47.8)	8(11.9)	.190
	6–7	107(47.6)	29(27.4)	59(55.7)	18(17)	
	<6	50(22.2)	15(30)	21(42)	14(28)	
Age ^b^; mean (SD) (year)		23.06(2.63)	22.85(2.61)	23.01(2.55)	23.68(2.90)	.264
Frequency of exercise ^b^; mean (SD) (Day/week)		2.03(2.13)	1.08(1.74)	2.18(2.05)	3.15(2.30)	< .001
Duration of exercise ^b^; mean(SD) (minutes)		32.58(37.76)	14.58(24.57)	33.66(33.05)	60.88(50.63)	< .001
BMI ^b^; mean(SD) (kg/m^2^)		23.86(3.87)	22.79(3.56)	24.41(3.98)	24.22(3.81)	.018
QOL ^b^		42.39(3.68)	42.31(3.37)	42.04(3.62)	43.44(4.25)	.123

^a^Chi-Square

bANOVA

sd: standard diviation

Pearson correlation test showed that PA (r = -0.726, P<0.001) and QOL (r = -0.405, P<0.001) exhibited significantly inverse relationship with BMI, whereas QOL and PA were proven to be positively related (r = 0.357, P<0.001) (Table 2).

**Table 2:** Correlation and internal consistency of the variables.

	Range	Cronbach alpha	Total MET physical	BMI	QOL
1.Total MET physical	0–16632	NA	1		
2.BMI	16.56–36.33	NA	-.726**	1	
3.QOL	34–55	.702	.357**	-.405*	1

* P < .05

**P < .01, MET = Metabolic Equivalents, BMI = Body Mass Index, QOL = Quality of life

The Table 3, indicates that the proposed fitting indices were relatively weak because of collinearity. In order to improve the fit of the proposed model, correlation of the residuals was employed. Correlation values greater than 4 were considered to be the correction indicator. The fitting for the suggested pattern was validated by the presented indices. The fitting indices for the final pattern prove that the fitting is valid.

**Table 3:** Fit indices of the primary and modified model.

	GFI	IFI	PCFI	CFI	PNEI	RMSEA	CMIN/df	P-Value	df	χ^2^
**Primary model**	.891	.835	.517	.819	.510	.097	3.113	< .001	53	165.012
**Modified model**	.992	.933	.574	.914	.520	.075	2.263	< .001	51	115.459

**Abbreviations;** CMIN/DF: Chi-square/degree-of-freedom ratio; RMSEA: Root Mean Square Error of Approximation; PCFI: Parsimonious Comparative Fit Index; GFI: Goodness of Fit Index; PNFI: Parsimonious Normed Fit Index; IFI: Incremental Fit Index; CFI: Comparative Fit Index. Fit indices: PNFI, PCFI (>.5), CFI, IFI, GFI (>.9), RMSEA (<0.05 good, 0.05–0.08 accept, 0.08–0.1 marginal), CMIN/DF (<3 good, <5 acceptable) [47].

)e.g.,χ^2^ = 115.459; RMSEA = .075; CFI = .914; IFI = .933; GFI = .992; PNFI = .520; PCFI = .914)

QOL can present 41% (R^2^ = .409) of the changes associated with physical activity and BMI in this model.

**Fig 1:**
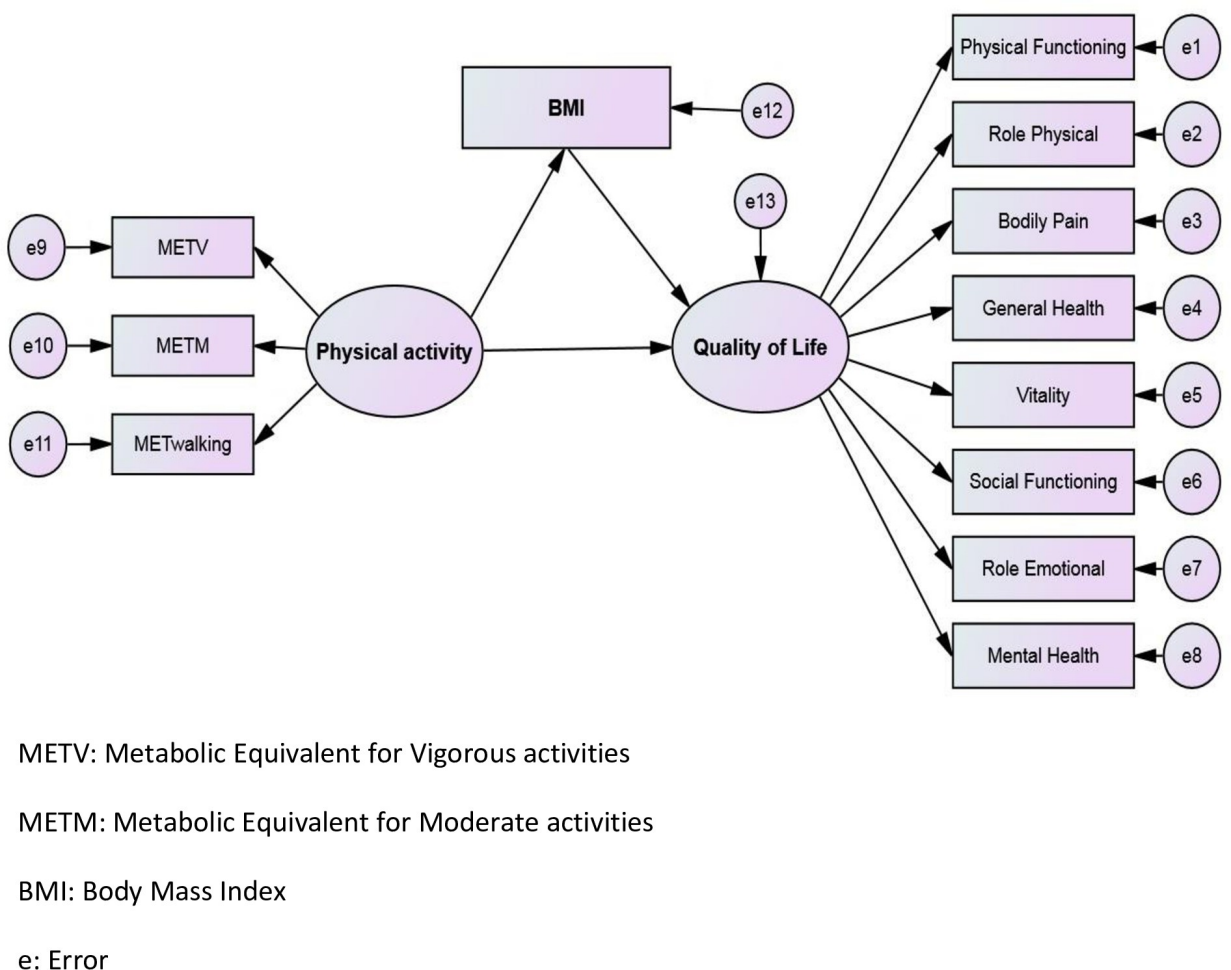
Hypothesized model of the variables (N = 225).

Fig 1 shows the standard coefficients of the research model.

The standardized regression coefficient showed that PA had a significant and inverse relationship with BMI (P < 0.001, β = -0.671) and a significant and direct relationship with QOL (P < 0.001, β = 0.501). In addition to these relations, BMI has also proven to be inversely related to QOL (P < 0.001, β = -0.396). Bootstrap testing showed BMI has a mediatory role in the relationship between PA and QOL (P < 0.001, Indirect Effects = 0.265) (Table 4).

**Table 4:** Standardized path coefficients of the mediation model.

Path	Estimate	S.E.	C.R.	P
BMI <—PA	.671-	0.017	-10.565	< .001
QOL <—PA	.501	.020	-8.342	< .001
QOL <—BMI	-.396	.028	6.557	< .001
	**Estimate**	**S.E.**	**95% CI**	**P**
Indirect Effects	.265	.067	.187,.334	< .001

PA = Physical activity, QOL = Quality of life, BMI = Body Mass Index, S.E = Standard Error, CI = Confidence Interval

**Fig 2:**
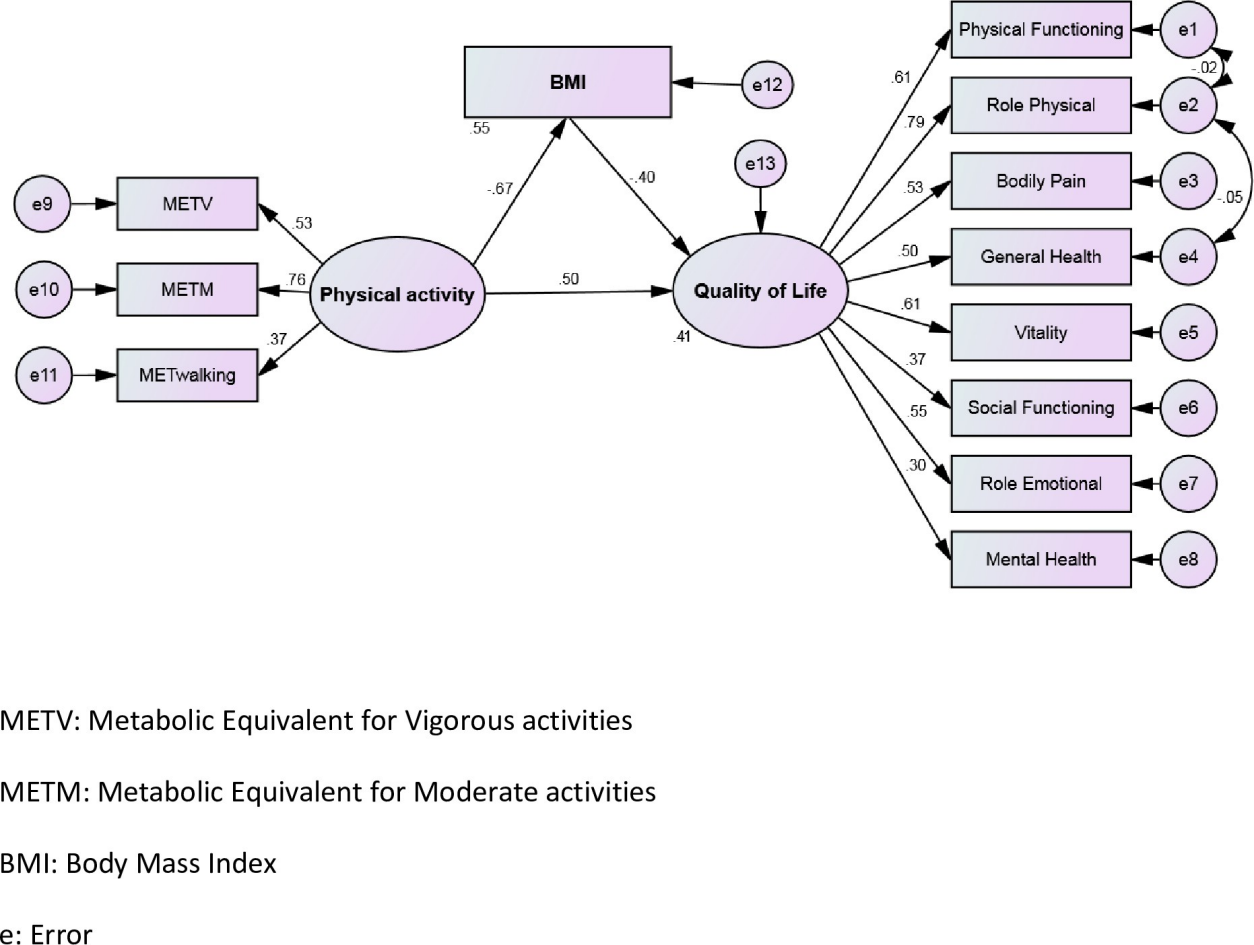
Standard coefficients of the modified model.

Path coefficients of the research model with respect to gender have been presented in Table 5. The fitting indices validate the proposed pattern in both genders, as shown in Table 5. It can be perceived that the effect of BMI on QOL was significant in men (P < 0.001, β = -0.307) in compare to women (P = 0.324, β = -0.158). The other paths however exhibit no difference between the two genders. The measured weight model (in two models unconstrained and constrained) results signify gender as a moderator in the relationship between physical activity and QOL (Δχ^2^ (10) = 19.903, P = 0.030) and also the mediatory role of BMI among students. In the modified model, by drawing a correlation between the errors (e1-e2 and e2-e4) the fit indices was improved (Fig 2).

**Table 5:** The effect of gender as a moderator and body mass index as a mediator for physical activity and quality of life among students.

Moderate		Path	Estimate	CMIN/df	RMSEA	CFI	GFI
Gender	Female	BMI <—PA	-.661*	2.759	.066	.910	.978
		QOL <—PA	.354*				
		QOL <—BMI	-.158				
		Indirect effect	.104*				
	Male	BMI <—PA	-.664*	2.768	.063	.908	.976
		QOL <—PA	.305*				
		QOL <—BMI	-.307*				
		Indirect effects	.203*				

* P < .01, PA = Physical activity, QOL = Quality of life, CMIN/DF = Chi-square/degree-of-freedom ratio, CFI = Comparative Fit Index. Fit indices, RMSEA = Root Mean Square Error of Approximation, GFI = Goodness of Fit Index

The results also showed that the indirect effect of BMI on physical activity and quality of life in men (β = 0.203, P < .001) was higher and more significant than in women (β = 0.104, P < .001). Other independent variables were not included in the model.

## Discussion

The study aimed to analyze the relationships among PA level, BMI, and gender in the QOL of Students.

The present study demonstrated that PA had a direct positive relationship with QOL, inverse relationship with BMI, and BMI had an inverse relationship with QOL. Furthermore, Physical activity had an indirect relationship with QOL as well. In addition, BMI mediated the influence of physical activity on QOL. Finally, gender had a moderator role in the relationships. The present study shows that physical activity is positively related to the QOL, which is in accordance with similar studies [8, 48, 49]. The hypothesis suggesting the predictability of QOL through physical activity with the mediatory role of BMI was proven to be true. The results are also in agreement with similar studies which supported the idea that the intensity of physical activity plays a significant role in QOL [50, 51]. It is reported that the motivation and adherence to physical activity are affected by multiple factors, such as age, sex, occupation, socioeconomic status and psychosocial state [49, 52].

We have also studied the relationship between PA levels and some other variables. We found a significant relationship between physical activity levels and gender, occupation, smoking, and BMI. A study by Cicek also suggests such a positive relationship which is in accordance with our results [53]. Furthermore, it was suggested that the intensity of physical activity was significantly affected by gender which is also in accordance with our results [53–55]. In the present study, the mean MET was different in different gender.

Contrary to the results of this research, another study suggests that the level of PA does not affect QOL [32]. However, this difference can be justified by the fact that the QOL is affected by multiple factors in older people and this suggests the importance of the age range of the participants.

In comparison with the gender, similar results have been reported in a study in Poland, which suggested that male adolescents are more active than females (32). Even more, studies can be found, verifying the higher activity levels in men (33).

We found a significant difference in BMI according to gender. Similar research in Turkey also reported a significant relationship between BMI and gender. It was perceived that males were at a higher risk for obesity, compared to females; these results are contrary to similar research that report a higher prevalence of obesity among females [56, 57]. The gender-related differences in our study can be attributed to Iranian culture such as eating outside and the increased eating of high-calorie food which is more prevalent among males than females.

The present study showed that a significant inverse relationship exists between physical activity and BMI; this is while the relationship between physical activity and QOL was seen to be direct. However, the PA was seen to be higher in male participants, despite higher BMI. The results of χ^2^ test signify a relationship between BMI and intensity of physical activity (P = 0.018).

While other studies suggest a poorer QOL in obese people [58, 59], the present study proves that BMI affects the QOL in men more than in women). The effect of BMI is significant in men and insignificant in women.

The present study proved BMI as a mediator in the relationship between PA and QOL. Similarly, the mediatory role of BMI was also seen in studies regarding children’s PA [60–63]. In a study by Bottcher et al., it was reported that weight-loss, leads to a diminish in the BMI, which in turn leads to a higher QOL score [64]. Although there are not many studies that discuss the relationship by considering a mediatory role, another study suggests that regular physical activity had a relationship with QOL with no effect from BMI values [65]. These contradictions could be due to the difference in the method of statistical analysis.

A research by Kim et al. shed light on the fact that the department of the study had a relationship with physical activity. Their work reported that students studying sports had a higher PA level and hence, better QOL scores [66].

The results indicate that most of the participants are in the normal BMI range. Student life invokes poor health habits, such as a reduction in physical activity [67].

The model suggests that health education programs focused on promoting regular PA and controlling BMI may be effective at improving QOL in young adults and college-aged populations. It can be said that the promotion of higher physical activity could prove to be beneficial to the QOL of college students. The moderating role of gender suggests an unhealthier lifestyle among college boys which could be due to the cultural practice in Iran.

The limitations of this study include the generalizability of the results to other samples and populations, and cross-sectional research conduction which may affect the relationship between the variables studied.

The implication of the research:

The results of this study could shed light on the possible factors, affecting the QOL. This information can be used by the health system to encourage higher levels of physical activity, culminating in a refined QOL.

## Conclusion

The present study showed that the PA levels among students of BUMS were relatively low. We assume that an improved PA through proper exercise, as well as changes in lifestyle, could serve as a method to improve the QOL in the population.
